# Annexin A1 Tripeptide Mimetic Increases Sirtuin-3 and Augments Mitochondrial Function to Limit Ischemic Kidney Injury

**DOI:** 10.3389/fphys.2021.683098

**Published:** 2021-07-01

**Authors:** Hagir Suliman, Qing Ma, Zhiquan Zhang, Jiafa Ren, Benjamin T. Morris, Steven D. Crowley, Luis Ulloa, Jamie R. Privratsky

**Affiliations:** ^1^Center for Perioperative Organ Protection, Department of Anesthesiology. Duke University Medical Center, Durham, NC, United States; ^2^Department of Medicine, Duke University Medical Center, Durham, NC, United States; ^3^Department of Medicine, Durham VA Medical Center, Durham, NC, United States

**Keywords:** acute kidney injury, mitochondrial integrity, sirtuin 3, ischemia/reperfusion injury, mitochondria

## Abstract

**Background:** Acute kidney injury (AKI) is one of the most common organ failures following surgery. We have developed a tripeptide mimetic (ANXA1sp) of the parent annexin A1 molecule that shows promise as an organ protectant limiting cellular stress; however, its potential as a kidney protective agent remains unexplored, and its mechanism of action is poorly understood. Our hypothesis was that ANXA1sp would limit kidney injury following surgical ischemic kidney injury.

**Methods:** In a blinded fashion, wildtype mice were assigned to receive vehicle control or ANXA1sp one hour prior to and one hour after kidney vascular clamping. Our primary outcomes were markers of kidney injury and function as measured by serum creatinine and histologic injury scoring of kidney tissue sections. Immunofluorescence microscopy, real-time PCR, and Western blot were used to assess cell death, oxidative stress, and mitochondrial biomarkers. An *in vitro* model of oxygen-glucose deprivation in immortalized kidney tubule cells was used.

**Results:** ANXA1sp given prior to and after ischemic kidney injury abrogated ischemic kidney injury. ANXA1sp limited cell death both *in vivo* and *in vitro* and abrogated oxidative stress following ischemia. ANXA1sp significantly increased the expression of markers associated with protective mitophagy and limited the expression of markers associated with detrimental mitochondrial fission. ANXA1sp upregulated the expression of the mitochondrial protectant sirtuin-3 (SIRT3) in the mitochondria of kidney tubular cells. Silencing of SIRT3 reversed ANXA1sp-mediated protection against hypoxic cell death.

**Conclusions:** ANXA1sp limits kidney injury, upregulates SIRT3, and preserves mitochondrial integrity following ischemic kidney injury. ANXA1sp holds considerable promise as a perioperative kidney protectant prior to ischemia inducing surgery and kidney transplantation.

## Introduction

Acute kidney injury (AKI) is one of the most common forms of organ injury occurring in up to 5% of all hospitalized patients, 10–30% of post-surgical patients (Zarbock et al., 2018), and 30% of critically ill patients (Thadhani et al., 1996). AKI increases morbidity and mortality and results in longer ICU and hospital stays, leading to increased hospital costs (Harris et al., 2015). Even small elevations in serum creatinine that do not meet the diagnostic criteria for AKI are associated with increased perioperative and long-term mortality (Lassnigg et al., 2004; Hobson et al., 2009; Kork et al., 2015). Despite its significant morbidity and mortality, there are currently no therapeutic modalities to prevent or treat AKI once it occurs. Thus, novel therapeutic modalities are needed.

Due to its high metabolic demands and oxygen consumption, the kidney is particularly susceptible to metabolic and oxidative stress (Bhargava and Schnellmann, 2017). Kidney tubule cells are rich in mitochondria that are required for efficient ATP production via oxidative phosphorylation (Bhargava and Schnellmann, 2017). Kidney tubular cells depend primarily on mitochondrial energy production making them sensitive to mitochondrial dysfunction, and mitochondrial impairment in the kidneys can severely affect kidney health (Gomez et al., 2015; Bhargava and Schnellmann, 2017). Several studies have suggested that mitochondrial damage and dysfunction contribute significantly to AKI development and impede kidney repair and regeneration (Tran et al., 2011, 2016). For example, mitochondrial fragmentation, swelling, and inner cristae loss were observed in experimental models of ischemic AKI (Xiao et al., 2014; Yang et al., 2014; Parikh et al., 2015) even prior to overt kidney cell apoptosis (Brooks et al., 2009). Mitochondria are central to the regulation of both cellular metabolism and the integration of pathways that lead to cell death within the kidney. As such, targeting mitochondrial quality control is a promising therapeutic target. In this regard, sirtuin-3 (SIRT3) is a mitochondrial NAD^+^ dependent deacetylase that maintains mitochondrial integrity under conditions of cellular stress (Perico et al., 2016; Hershberger et al., 2017; Marcus and Andrabi, 2018). In addition, SIRT3 has been shown to protect against toxic (Morigi et al., 2015) and septic (Zhao et al., 2018) AKI. Developing therapeutic agents that upregulate SIRT3 and protect the mitochondria could have broad implications for kidney protection prior to AKI-inducing stimuli (i.e., surgery, transplantation) and during recovery following AKI.

Annexin A1 is a 37 kD endogenous protein that is expressed mainly by immune cells and epithelial cells (Leoni and Nusrat, 2016). Annexin A1 is a well-established pro-resolving, anti-inflammatory mediator (Gavins and Hickey, 2012; Leoni and Nusrat, 2016). As a result, peptide fragments of this molecule have been generated and shown to have protective anti-inflammatory properties in many disease states (Sugimoto et al., 2016), including in kidney ischemia/reperfusion injury in rats (Facio et al., 2011). Our group has developed a specific small tripeptide fragment of the human annexin A1 molecule (ANXA1sp) that exerts potent anti-inflammatory properties (Zhang et al., 2010) and upregulates SIRT3 in the brain (Ma et al., 2019). Based upon the promising protective role of SIRT3 in toxic and inflammatory AKI (Morigi et al., 2015; Zhao et al., 2018), we hypothesized that ANXA1sp would protect against ischemic AKI through the upregulation of SIRT3, mitochondrial protection, and amelioration of tubular cell death. Here, we analyze how ANXA1sp treatment affects kidney injury, mitochondrial function, SIRT3 levels, and cell death following ischemic AKI. These studies have important implications for kidney protection during surgery and prior to kidney transplantation.

## Methods

### Chemicals and Reagents

Annexin A1 tripeptide fragment (Ac-Gln-Ala-Trp) (ANXA1sp) was synthesized by GenScript Biotech (Piscataway, NJ) as previously described (Zhang et al., 2010) and was reconstituted in DMSO and placed in individual doses. Ketamine, xylazine, and buprenorphine were purchased from Henry Schein animal health (Dublin, OH).

### Animal Experiments

All of the animal studies were approved by the Durham Veterans Affairs Medical Center (VAMC) Institutional Animal Care and Use Committee, performed at the Durham VAMC, and conducted in accordance with the National Institutes of Health Guide for the Care and Use of Laboratory Animals. Briefly, 129/SvEv 10–16-week-old male mice were obtained from Taconic Biosciences (Rensselaer, NY). Mice were fed a standard chow diet.

#### Administration of ANXA1sp

Mice were randomly assigned to receive either the vehicle control or experimental drug. The investigators performing surgery, experiments, and analyzing the data were blinded to the treatment groups. Both DMSO Vehicle and ANXA1sp doses were reconstituted in saline and at one hour prior to clamp placement, 1 mg/kg was given intraperitoneally (IP). The same treatment was given at 1-h post-clamp removal.

#### Ischemia/Reperfusion (I/R)

I/R protocol was based on the procedure by Skrypnyk et al., 2013. We used a unilateral ischemia with a contralateral nephrectomy model to avoid uneven clamp pressures and variable responses to ischemia between kidneys, which limits variability. Briefly, mice were anesthetized with ketamine (120 mg/kg)/xylazine (12 mg/kg). Mice were placed on a warming pad (Hallowell EMC, Pittsfield, MA) heated to 38°C by a Gaymar TP650 water pump. After aseptic prep, a midline dorsal incision was created, and blunt dissection was performed toward the right kidney. The flank muscle and fascia above the right kidney was incised and the right kidney was exteriorized; after which, the renal pedicle was ligated with suture and the right kidney was removed. After the closure of fascia and muscle over the right kidney, blunt dissection was performed toward the left kidney. The flank muscle and fascia above the left kidney were incised, and the left kidney was exteriorized. Adipose and connective tissue were carefully removed near the renal vessels and an 800 g pressure clamp (Fine Science Tools) was placed on the left renal pedicle for 33 min. The mouse was covered with a sterile foil covering (Braintree Scientific, Inc., Braintree, MA; cat#SPDR-MPS) until the clamp was removed. Our group has found that maintaining mice on a 38°C heating pad with foil covering maintains the mouse rectal temperature between 34.5 and 36 C for the duration of surgery to the same extent of feedback temperature systems while avoiding large environmental temperature fluctuations between animals. At the end of ischemic time, the clamp was removed and reperfusion was confirmed by the color change in the kidney. The fascia and muscle layer and skin were closed by suture. Our approved perioperative pain control regimen is as follows: acetaminophen (300 mg/kg/d) is placed in drinking water two days prior to surgery and up to four days after surgery or until harvest, whichever is first; the incision is infiltrated with bupivacaine (0.25%) prior to skin closure; and mice are injected once with buprenorphine (0.1 mcg/gm) immediately after surgery.

### Blood and Serum Analyses

Blood was collected from a cardiac puncture at the indicated time points, allowed to clot for 30 min at room temperature, and centrifuged at 3,000 g for 10 min at 4°C. Serum creatinine levels were measured by the Jaffe method by the Animal Histopathology and Laboratory Medicine Core at the University of North Carolina, which is supported in part by an NCI Center Core Support Grant (5P30CA016086-41) to the UNC Lineberger Comprehensive Cancer Center.

### Histologic Analyses

Kidney tissues were removed and a cross-sectional segment was obtained. The kidney segment was fixed with 10% neutral-buffered formalin (VWR 16004-128), embedded with paraffin, sectioned in 5-μm sections by the Duke Research Immunohistology Laboratory.

#### Injury Scoring

Sections were stained with periodic acid Schiff (PAS) staining and scored by an experienced animal pathologist masked to experimental groups. Sections were graded according to a previously established scoring system (Zhang et al., 2014): the percentages of tubules with cell lysis, loss of brush, border, and cast formation were scored on a scale from 0 to 4 (0, no damage; 1, 25%; 2, 25%−50%; 3, 50%−75%; 4, >75%). Histologic scores for each kidney were obtained by adding the individual component scores.

#### 8-OHdG and Citrate Synthase Immunofluorescence Staining

Paraffin-embedded kidney sections (4 μm) were processed for immunostaining by deparaffinization in xylene and rehydration through a descending series of alcohols, washed extensively in 0.1 M PBS, blocked with 10% normal goat serum, and incubated in primary antibodies to 8-hydroxy-2'deoxyguanosine (8-OHdG) (Abcam Cat# ab48508, RRID:AB_867461) or citrate synthase (GeneTex Cat# GTX110624, RRID:AB_1950045) diluted in 10% normal goat serum overnight at 4°C, followed by fluorescent-labeled secondary antibodies labeled with Alexa Fluor 488 green or Alexa Fluor 556 red. Nuclei were counterstained with DAPI. Images were acquired on a Nikon E400 fluorescence microscope.

#### LC3 and Citrate Synthase Immunofluorescence Staining

Kidney samples were paraffin-embedded, cut into 5-μm sections, and mounted on slides. Three channel immunofluorescence staining were performed for mitochondrial citrate synthase (CS; primary antibody; 1:200; GeneTex Cat# GTX110624, RRID:AB_1950045), mitophagy marker LC3 (Santa Cruz Biotechnology Cat# sc-54237, RRID:AB_2137716), and DAPI (Molecular Probes) for nuclei. Alexa-Fluor-coupled secondary antibodies (Invitrogen) were used at 1:400. Stained sections were examined on a Zeiss LSM 710 laser-scanning confocal microscope to identify and localize mitophagosomes. The colocalization was assessed by three-channel fluorescence: green (for CS), red (for LC3), and blue (DAPI).

#### SIRT3 and Mitochondrial Complex IV Immunofluorescence Staining

After deparaffinization, kidney tissue sections were treated with 10 mM citrate buffer (pH 6.0) for antigen retrieval. After blocking with 10% normal goat serum and 0.1% BSA at RT for one hour, the sections were incubated with rabbit anti-SIRT3 (1:300; Cell Signaling Technology Cat# 5490, RRID:AB_10828246) and mouse anti-COXIV (1:500; Santa Cruz Biotechnology) at 4°C overnight. The sections were then incubated with Alexa Fluor 488-conjugated goat anti-rabbit IgG (1:500; Invitrogen, Carlsbad, CA, United States) and Alexa Fluor 550-conjugated goat anti-mouse IgG (1:500; Invitrogen, Carlsbad, CA, United States) at RT for one hour. After washing with PBS, slides were prepared and mounted using the UltraCruz™ Mounting Medium with DAPI (Santa Cruz Biotechnology, Santa Cruz, CA, United States) to detect nuclei. Images were captured on a Leica fluorescent microscope (Leica DM IRB, Germany) using a 20X/0.4 PH objective at 1.5-fold magnification, and the images were analyzed by the NIH ImageJ software (version 1.51).

### RT-PCR

mRNA was isolated with a RNeasy Mini Kit (Qiagen, Germantown, MD) per kit instructions. A cross-sectional piece of kidney containing cortex and medulla was homogenized in Buffer RLT with 0.01% β-ME and further homogenized with Qiashredder columns (Qiagen 79654). mRNA concentration was measured by Nanodrop (ThermoFisher). The High-Capacity cDNA Reverse Transcription Kit (Applied Biosystems 4368814) was used to synthesize cDNA according to the manufacturer's instructions. Gene expression levels of SIRT3 was determined by RT-PCR on an ABI7900HT machine (Applied Biosystems) using TaqMan primers to SIRT3 (mouse: Mm00452131_m1, #4331182—ThermoFisher; human: Hs00202030_m1, # 4331182—ThermoFisher).

### Western Blot

A piece from flash frozen kidneys were homogenized in RIPA buffer containing cocktail protease and phosphatase inhibitors. Total protein content was measured by the BCA assay. In total, 20 μg of total protein was loaded into SDS-PAGE gels, and immunoblots were performed as previously described (Suliman et al., 2007). Antibodies used include Pink1 (1:500; Abcam Cat# ab23707, RRID:AB_447627), Parkin (1:500; Santa Cruz Biotechnology Cat# sc-32282, RRID:AB_628104), tubulin (1:1,000; Sigma-Aldrich Cat# T5168, RRID:AB_477579), mitochondrial porin (1:500; Santa Cruz Biotechnology Cat# sc-8829, RRID:AB_2214801), SOD2 (Abcam Cat# ab13533, RRID:AB_300434), Ogg1 (Novus Cat# NB100-106, RRID:AB_10104097), Drp1 (Cell Signaling Technology Cat# 8570, RRID:AB_10950498), SIRT3 (Cell Signaling Technology Cat# 5490, RRID:AB_10828246), LC3A/B (Cell Signaling Technology Cat#4108, RRID:AB_2137703), mitochondrial transcription factor A (Tfam) (Abcam Cat# ab131607, RRID:AB_11154693), citrate synthase (Millipore Cat # MAB3087, RRID:AB_2084760), mtCI (total OXPHOS, Abcam Cat# ab110413, RRID:AB_2629281), and ND-1 (Santa Cruz Biotechnology Cat #sc-293243).

#### Immunoprecipitation and Acetylation Analysis

Kidney tissue lysate (0.5 mg) was pre-cleaned with 20 μl of A/G plus agarose (Cat. No. sc-2003; Santa Cruz Biotechnology) and precipitated by 20 μl of anti-SOD2 (Abcam Cat# ab13533, RRID:AB_300434) at 4°C overnight. Following further precipitation with 20 μl of A/G plus agarose for 2 h, samples were washed three times using cold PBS, solubilized with 40 μl 2 × SDS sample buffer (Cat. No. S3401–1VL; Sigma-Aldrich). An equal amount of eluted protein was resolved on the gel for assessing the acetylation status SOD2 using anti-Ac-lysine (AKL5C1) antibody (Santa Cruz Biotechnology, Cat# sc-32268, RRID:AB_627898).

### Cell Culture

#### HK-2 Cell Culture

The human immortalized proximal tubule epithelial cell line HK-2 (ATCC, CRL-2190) was kindly provided by Dr. Tomokazu Souma and cultured in DMEM/F12 supplemented with 10%FBS, 1% penicillin/streptomycin, and 1% Insulin-Transferrin-Selenium solution (Gibco). The human RPTEC line immortalized with pLXSN-hTERT retroviral transfection (RPTEC/TERT1– ATCC, CRL-4031) was kindly provided by Dr. Alex Grabner and cultured in the REGM Renal Epithelial Growth Medium Bullet Kit (cat# CC-3190, Lonza).

#### Hypoxia Exposure

Cells were exposed to ANXA1sp (10, 20 μM) for 1 h. Cells were then subjected to oxygen–glucose deprivation (OGD: DMEM no glucose, 92% N2/3% H2/5% CO2) in an anaerobic chamber (Coy Laboratories) for timepoints indicated in the figure legends. Cells were lifted and analyzed for apoptosis/cell death per below. Normoxic cells were treated with DMEM + F12 with no serum for HK-2 cells and basal REGM media for RPTEC in a 37°C growth incubator with 95% air/5% CO2. Vehicle cells were treated with equal volumes of DMSO in the medium. Cells were harvested for further analysis.

### Cell Death Analysis

#### TUNEL Staining

Apoptosis was determined by terminal deoxynucleotidyl nick-end labeling (TUNEL) per assay manufacturer's protocol (Roche Diagnostics, Indianapolis, IN, United States). Paraffin-embedded sections (5 μm thick) were deparaffinized using xylene and descending grades of ethanol and incubated in a proteinase K working solution for 15–30 min at room temperature. Sections were then incubated with terminal deoxynucleotidyl transferase (TdT) for 1 h at 37°C and then rinsed with PBS. Slides were counterstained with DAPI and cover slipped using the Dako Fluorescence Mounting Media (Agilent Technologies, Santa Clara, CA). For each section, five areas of the corticomedullary junction were imaged at 20X; images were post-processed within Image J with intensity parameters equal amongst all images; and nuclei were scored as positive if there was DAPI and TUNEL staining positivity in the same location. TUNEL positivity for each animal was reported as the number of TUNEL positive nuclei per 20X field averaged over the five images.

#### Cell Death Assay

HK-2 and RPTEC were treated with ANXA1sp and hypoxia per above. At the end of experiment, the supernatant was removed to collect dead cells, cells lifted with TrypLE (Gibco), TrypLE inactivated with 10% FBS, and cellular fractions combined with supernatants. Cells were spun down (350 g for 5 min), and pellet was resuspended in 100 μl media. Apoptosis was determined by Muse Annexin V & Dead Cell Kit (Luminex) per kit instructions.

### Statistical Analyses

Statistical tests were performed with the GraphPad Prism Software® (GraphPad Software, La Jolla, CA). The figures are representative of the experiments that were repeated at least twice on different days, and the data are expressed as mean ± standard error of the mean (SEM). Comparisons with only two groups were analyzed by unpaired *T* test. Comparisons with more than two groups were analyzed by one-way or two-way ANOVA as indicated in each figure legend with the Sidak's multiple comparisons test for *post-hoc* analysis to compare groups.

## Results

### ANXA1sp Tripeptide Prevents Kidney Injury Following Ischemia

**Figure 1:**
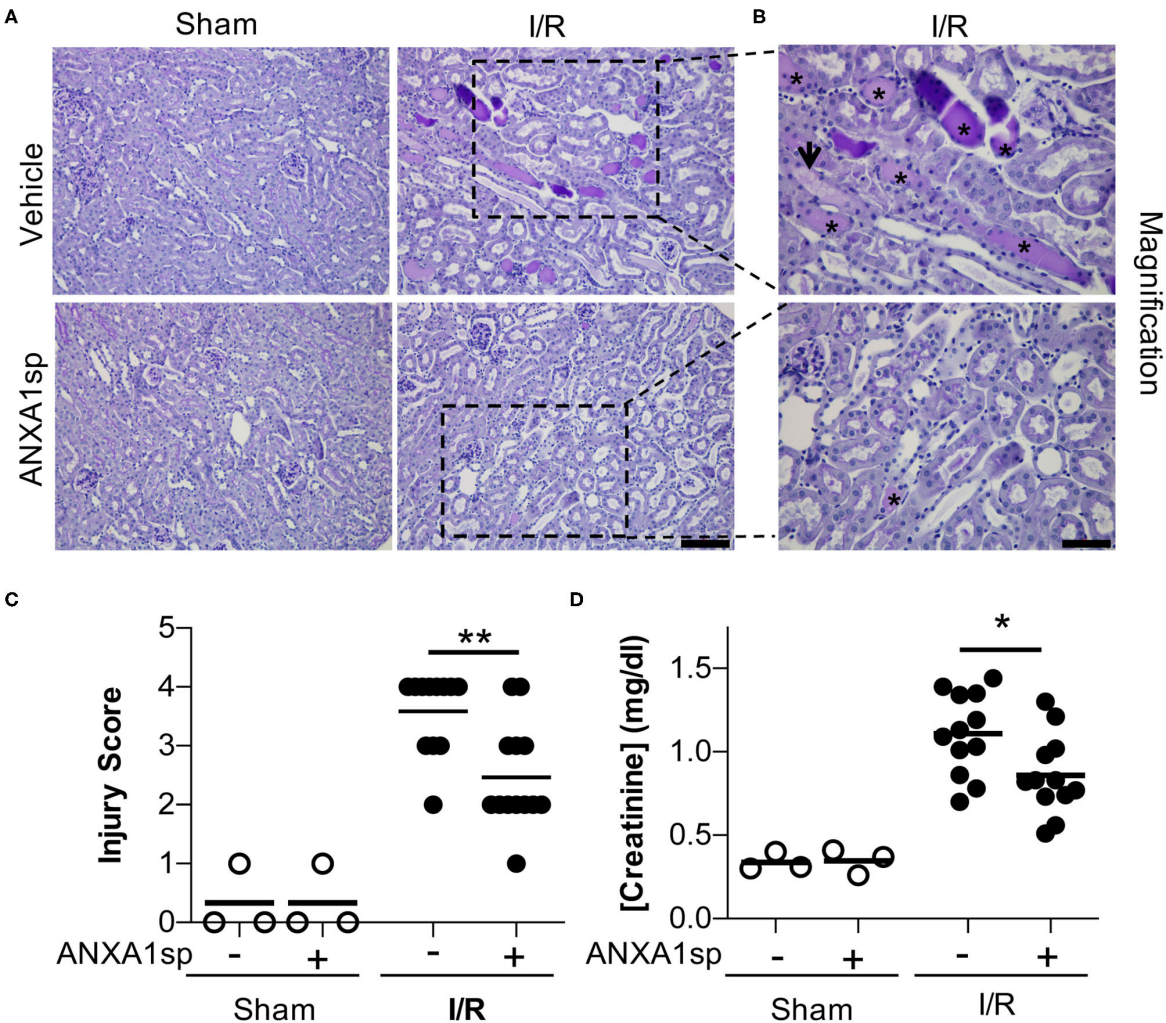
AnnexinA1 tripeptide (ANXA1sp) attenuates kidney injury following ischemia/reperfusion-induced kidney injury. Mice were treated with either Vehicle or ANXA1sp and subjected to 33 min of unilateral ischemia and contralateral nephrectomy and then re-injected with Vehicle or ANXA1sp 1 h after reperfusion. **(A)** Representative periodic-acid Schiff (PAS)-stained kidney sections demonstrating increased injury in Vehicle, I/R group at Day 1 after ischemia (*scale bar* = 100 um). **(B)** Increased magnification of boxes in **(A)** to demonstrate increased histologic evidence of injury in Vehicle, I/R mice (asterisk: protein casts; arrowhead: tubule vacuolization) compared to ANXA1sp-treated mice (*scale bar* = 50 um). **(C)** Histologic injury scoring from **(A)** by observer blinded to experimental grouping (*n* = 3 for Sham groups, *n* = 12 for I/R groups). ANXA1sp-treated mice display attenuated kidney injury. Statistical significance determined by two-way ANOVA with Sidak post-test (**p < 0.01). (**D**) Serum creatinine was measured. ANXA1sp-treated mice display ameliorated AKI compared to Vehicle-treated mice (*n* = 3 for Sham groups, *n* = 12 for I/R groups). Line on graph displays mean. Statistical significance determined by two-way ANOVA with Sidak post-test (^*^p < 0.05).

Annexin A1 is a pro-resolving mediator (Gavins and Hickey, 2012; Leoni and Nusrat, 2016), and peptide fragments of annexin A1 have been generated and shown to have protective properties in many disease states (Sugimoto et al., 2016). Here, we use a synthetic tripeptide of the N-terminal domain of the annexin A1 molecule (ANXA1sp) that retains the most potent anti-inflammatory and pro-resolving properties (Zhang et al., 2010) in the setting of ischemic kidney injury to determine its effect on kidney protection. We hypothesized that pre-treatment of mice with ANXA1sp would dampen the severity of AKI following ischemia/reperfusion (I/R). We injected mice with either vehicle or ANXA1sp one hour prior to ischemia, induced ischemia, and then injected mice with the same treatment one hour after reperfusion. Following I/R, we noted significantly less tubular injury (Figures 1A–C) and lower levels of serum creatinine (Figure 1D) at 24 h in the ANXA1sp-treated mice. We concluded that ANXA1sp treatment ameliorated ischemic kidney injury.

### ANXA1sp Prevents Kidney Tubule Apoptosis/Cell Death

**Figure 2:**
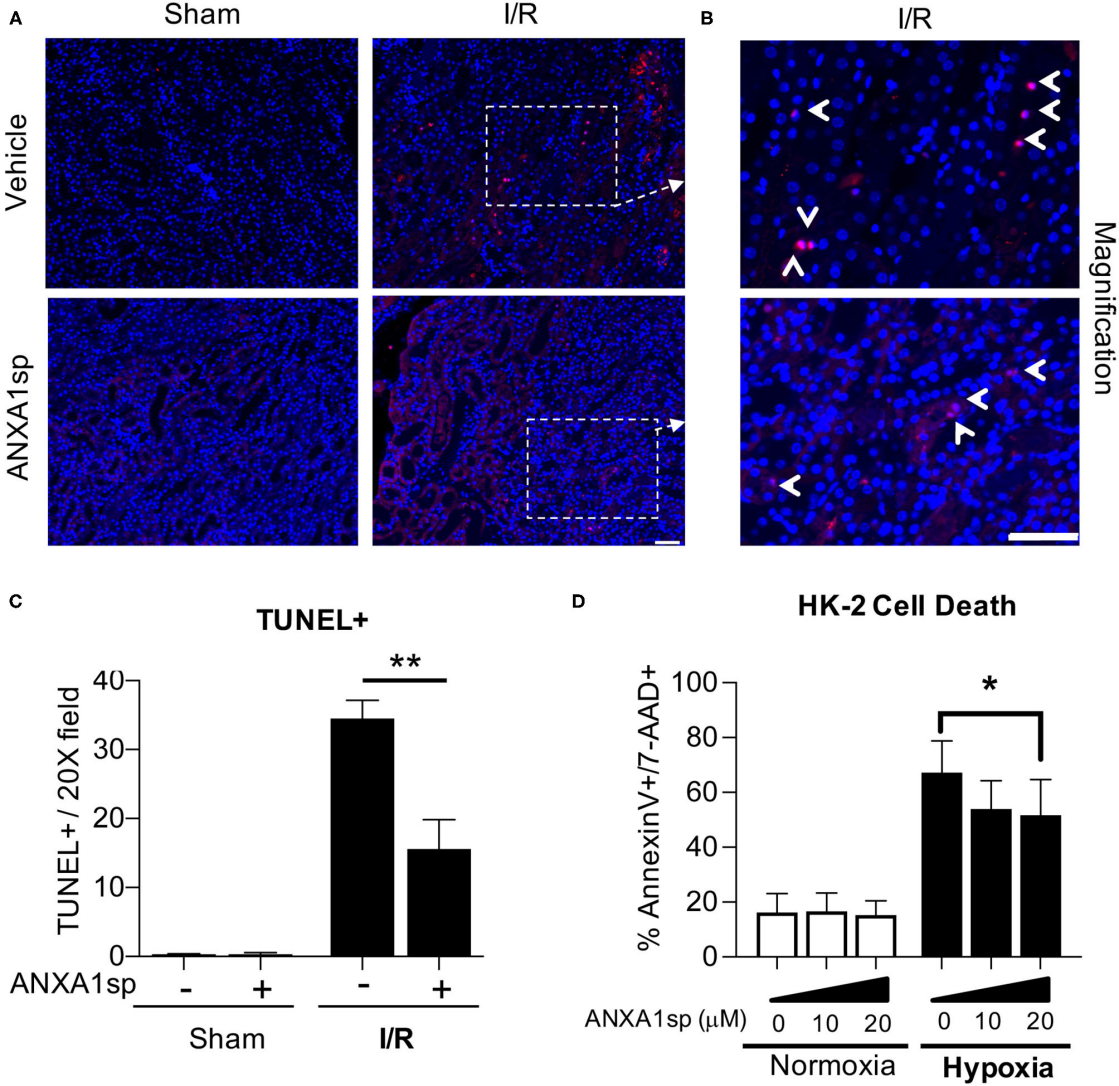
ANXA1sp treatment prevents kidney tubular cell death. Mice were treated with either Vehicle or ANXA1sp 1 h prior to ischemia, subjected to 33 minutes of unilateral ischemia and contralateral nephrectomy and then re-injected with Vehicle or ANXA1sp 1 h after reperfusion. **(A**) Kidney tissues were harvested at 24 h after reperfusion. Representative terminal deoxynucleotidyl transferase dUTP nick end labeling (TUNEL)-stained kidney sections demonstrating increased apoptosis in Vehicle, I/R group at Day 1 after ischemia. Scale bar shows 25 μm. **(B)** Increased magnification of boxes in **(A)** to demonstrate increased evidence of apoptosis in Vehicle, I/R mice (arrowhead: TUNEL-positive nuclei) compared to ANXA1sp-treated mice. Scale bar shows 25 μm. **(C)** Quantification of TUNEL positive nuclei from **(A)**. Graph displays mean +/- SEM of % TUNEL positive nuclei from five fields/section from each mouse (*n* = 3 for Sham and I/R groups) with significance determined by two-way ANOVA with Sidak post-test (^**^p < 0.01). (**D**) The immortalized human kidney cell line, HK-2, was grown to confluence in monolayers. Cells were pretreated with Vehicle or ANXA1sp at increasing concentrations and then subjected to 16 h of oxygen-glucose deprivation (hypoxia) in an anaerobic chamber. ANXA1sp prevented hypoxic cell death (*n* = 5–6/condition). Graphs display mean +/- SEM with significance determined by two-way ANOVA with Sidak post-test (^*^p < 0.05).

To examine the possible mechanisms of ANXA1sp-mediated kidney protection, we next wanted to determine whether ANXA1sp prevented cell death in the kidney following ischemia. We performed TUNEL staining on kidney sections following severe ischemia. We found that kidneys from the ANXA1sp-treated mice had fewer TUNEL-positive nuclei following I/R (Figures 2A–C). These results indicate that ANXA1sp is able to ameliorate cell death in the kidney following ischemic injury. Based on these findings, we then wanted to determine the effects of ANXA1sp on kidney tubular cell death *in vitro*. We developed a cellular model of ischemic kidney injury to mimic our *in vivo* model. We treated an immortalized human kidney epithelial cell line, HK-2, with vehicle or ANXA1sp and subjected cells to hypoxia using oxygen-glucose deprivation in an anaerobic chamber. We found that ANXA1sp given prior to hypoxia exposure was able to prevent hypoxic HK-2 cell death in a dose-dependent manner (Figure 2D) and hypoxic RPTEC cell death (Supplementary Figure 1). From these data, we concluded that ANXA1sp limits cell death in kidney tubular cells following ischemia.

### ANXA1sp Treatment Limits Oxidative Damage and Improves Mitochondrial Integrity

**Figure 3:**
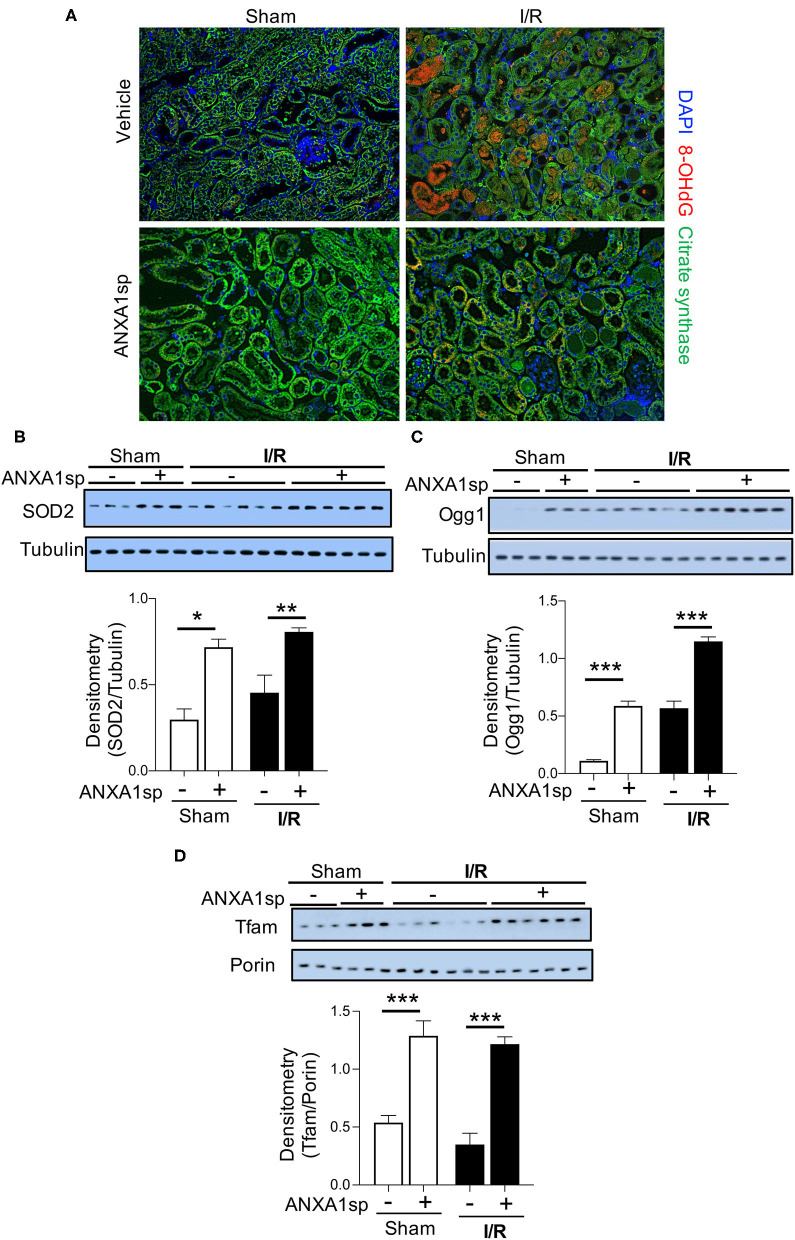
ANXA1sp treatment limits oxidative damage and improves markers of mitochondrial integrity and function. Mice were treated with either Vehicle or ANXA1sp 1 h prior to ischemia, subjected to 33 min of unilateral ischemia and contralateral nephrectomy, and then re-injected with Vehicle or ANXA1sp 1 h after reperfusion. Kidney tissues were harvested at 24 h after reperfusion. **(A)** Representative immunofluorescence histologic staining for 8-OHdG and citrate synthase. **(B)** Protein levels of superoxide dismutase 2 (SOD2) were determined by Western blot with densitometry shown in the graph below. **(C)** Protein levels of 8-Oxoguanine DNA Glycosylase (Ogg1) were determined by Western blot with densitometry shown in the graph below. **(D)** Mitochondrial protein levels of mitochondrial transcription factor A (Tfam) were determined by Western blot with densitometry shown in the graph below. Graphs display mean +/-SEM of densitometry of protein normalized to the loading control. Statistical significance determined by two-way ANOVA with Sidak post-test (*n* = 3 samples for Sham groups, n = 6 samples for I/R groups; ^*^p < 0.05, ^**^p < 0.01, ^***^p < 0.001).

Reactive oxygen species (ROS) are critical triggers of mitochondrial damage and can subsequently cause cell death. The 8-OHdG adduct is a product of mitochondrial (mt)DNA oxidation, and if not repaired, may lead to mutations and a dysfunctional mtDNA genome. We evaluated 8-OHdG levels in the kidney by immunofluorescence staining. Compared to sham animals, we found that 8-OHdG levels increased following ischemia (Figure 3A—compare sham to I/R panels). We found ROS staining to be localized to mitochondria as demonstrated by co-localization with citrate synthase (green) staining (Figure 3A). Following ischemia, ANXA1sp decreased the intensity of 8-OHdG staining (Figure 3A—bottom right) compared to vehicle treated mice (Figure 3A—top right), indicating that ANXA1sp limited accumulation of ROS following ischemia. In support of this notion, we found that ANXA1sp significantly increased the protein levels of the antioxidant enzyme superoxide dismutase 2 (SOD2) under both sham and ischemia conditions (Figure 3B), indicating that ANXA1sp is able to upregulate protective antioxidant enzymes. To further evaluate the integrity of the cellular machinery involved in maintaining the mitochondrial genome, we examined changes in protein expression of the mtDNA base-excision repair enzyme OGG1. ANXA1sp significantly increased the levels of kidney OGG1 protein expression compared to vehicle treatment (Figure 3C). Moreover, ANXA1sp upregulated the levels of mitochondrial transcription factor A (Tfam) (Figure 3D), indicating augmented mtDNA transcription and replication. Taken together, ANXA1sp limits markers of oxidative stress and upregulates mtDNA repair proteins and transcription factors as possible mechanisms of cellular protection and improved mitochondrial function.

### ANXA1sp Treatment Improves Markers of Mitophagy and Limits Markers of Mitochondrial Fission

**Figure 4:**
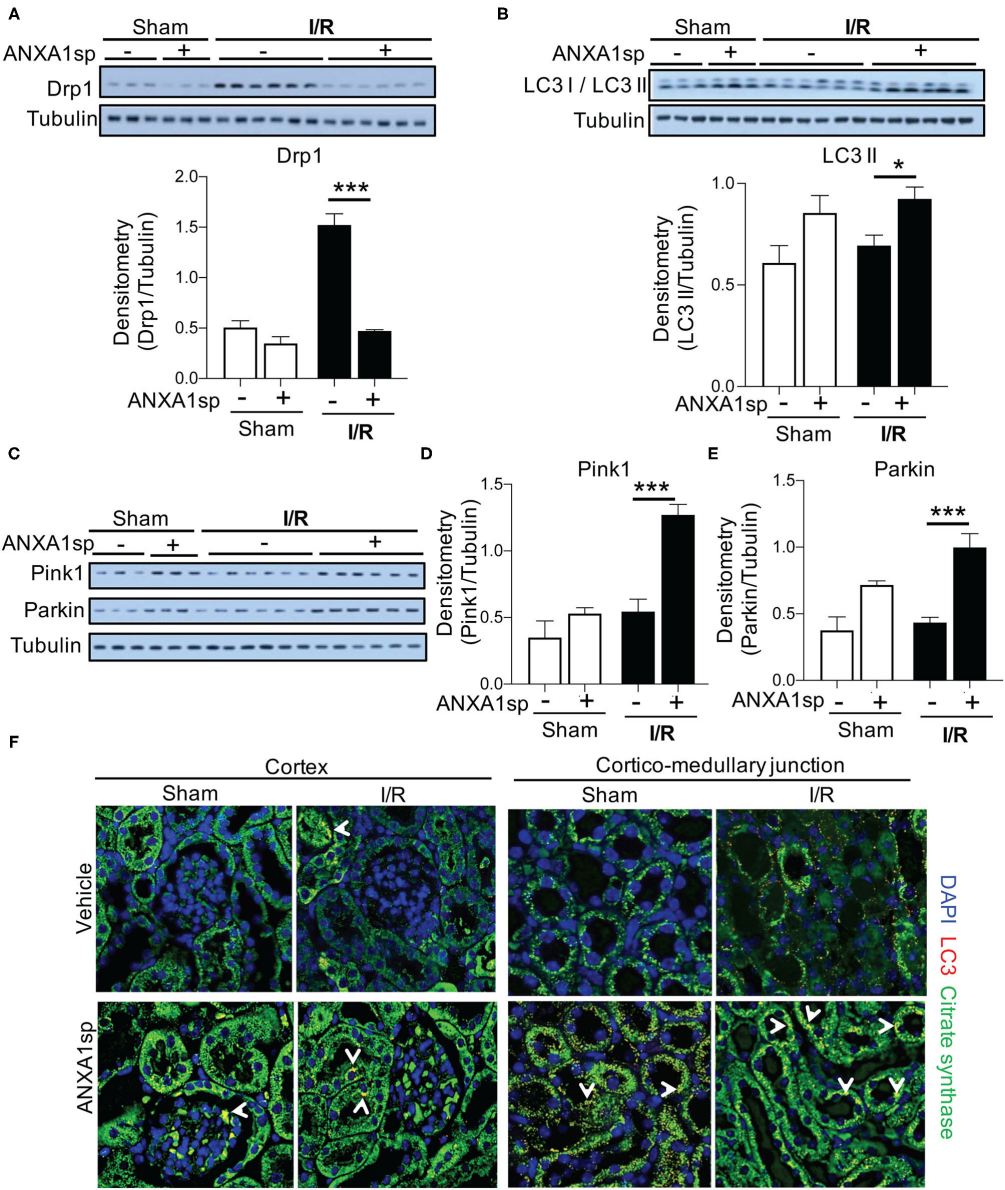
ANXA1sp treatment improves markers of mitochondrial integrity. Mice were treated with either Vehicle or ANXA1sp 1 h prior to ischemia, subjected to 33 min of unilateral ischemia and contralateral nephrectomy, and then re-injected with Vehicle or ANXA1sp 1 h after reperfusion. Kidney tissues were harvested at 24 h after reperfusion. **(A)** Protein levels of dynamin-related protein (Drp)1 were determined by Western blot with densitometry shown in the graph below. **(B)** Western blot for LC3 I/LC3 II showing protein levels of indicated proteins with densitometry shown in the graph below. **(C)** Western blot for Pink1 and Parkin with densitometry shown in **(D)** and **(E)**, respectively. Graphs display mean +/-SEM of densitometry of protein normalized to tubulin. Statistical significance determined by two-way ANOVA with Sidak post-test (*n* = 3 samples for Sham groups, *n* = 6 samples for I/R groups; ^*^p < 0.05, ^***^p < 0.001). **(F)** Confocal immunofluorescence micrographs of the kidney cortex (left panels) and corticomedullary junction (right panels). Images show confocal micrographs of kidney sections stained for LC3 (red), citrate synthase (green), and nuclei are counterstained with DAPI. Arrows denote overlay of LC3 and citrate synthase (yellow/orange fluorescence). Note the increased citrate synthase expression and LC3 puncta in corticomedullary region of ANXA1sp-treated mice. *Magnification* = 600x. Data are representative of three sets of mice.

As mitochondrial and oxidative stress can promote mitochondrial fragmentation and limit function (Srinivasan et al., 2017), we examined the impact of ischemia on mitochondrial morphology by measuring Drp1, which is expressed in the cytosol and is recruited to mitochondria undergoing fragmentation or damage. Following ischemia, kidneys from vehicle-treated mice showed a significant increase in Drp1 protein, which was completely abrogated by ANXA1sp treatment (Figure 4A). Along these lines, the elimination of damaged mitochondria through a process termed mitophagy is vital to maintaining cellular function in the face of cellular stress (Ploumi et al., 2017). ANXA1sp induced an accumulation of the lipidated form of microtubule associated protein 1 light chain 3 beta (LC3 II/LC3 I), a marker for autophagy and mitophagy (Figure 4B). We next determined the effect of ANXA1sp specifically on mitophagy following ischemic AKI. We assessed the levels of mitophagy regulators PINK1 (PTEN induced putative kinase 1) and Parkin. ANXA1sp treatment significantly increased both PINK1 and Parkin levels following ischemia (Figures 4C–E). Immunofluorescence staining further demonstrated that ANXA1sp treatment increased LC3 puncta colocalized to mitochondria especially in the corticomedullary region (Figure 4F), a region especially susceptible to ischemic injury due to its high metabolic demands. Taken together, we concluded that ANXA1sp improves mitochondrial integrity and promotes mitophagy as a mechanism of kidney protection.

### ANXA1sp Induces PGC1α-Mediated Mitochondrial Biogenesis in the Kidneys

To gain further insight into the potential impact of ANXA1sp on mitochondrial function following IR, and to determine whether ANXA1sp-mediated mitochondrial integrity is coordinated with mitochondrial biogenesis, mitochondrial biogenesis was tracked in mouse kidneys by immunoblotting for key mitochondrial proteins. Following ischemia, we found a significant upregulation of the major regulator of mitochondrial biogenesis, PGC1-α (Tran et al., 2011, 2016), in ANXA1sp-treated mice compared to vehicle-treated mice (Supplementary Figures 2A,B). As additional evidence of improved mitochondrial biogenesis following ANXA1sp treatment, compared to vehicle, ANXA1sp treatment increased mitochondrial mass in the kidney as measured by citrate synthase (mtCS) (Supplementary Figures 2C,D), cytochrome c oxidase subunit 1 of mitochondrial Complex IV (mtCI) (Supplementary Figures 2C,E), and the mitochondrial NADH-ubiquinone oxidoreductase chain 1 (mtND1) (Supplementary Figures 2C,F). Thus, ANXA1sp increases PGC1α, which upregulates key proteins involved in mitochondrial biogenesis, further supporting its role in mitochondrial protection following ischemic AKI.

### ANXA1sp Upregulates Sirtuin-3 (SIRT3) Following Ischemia

**Figure 5:**
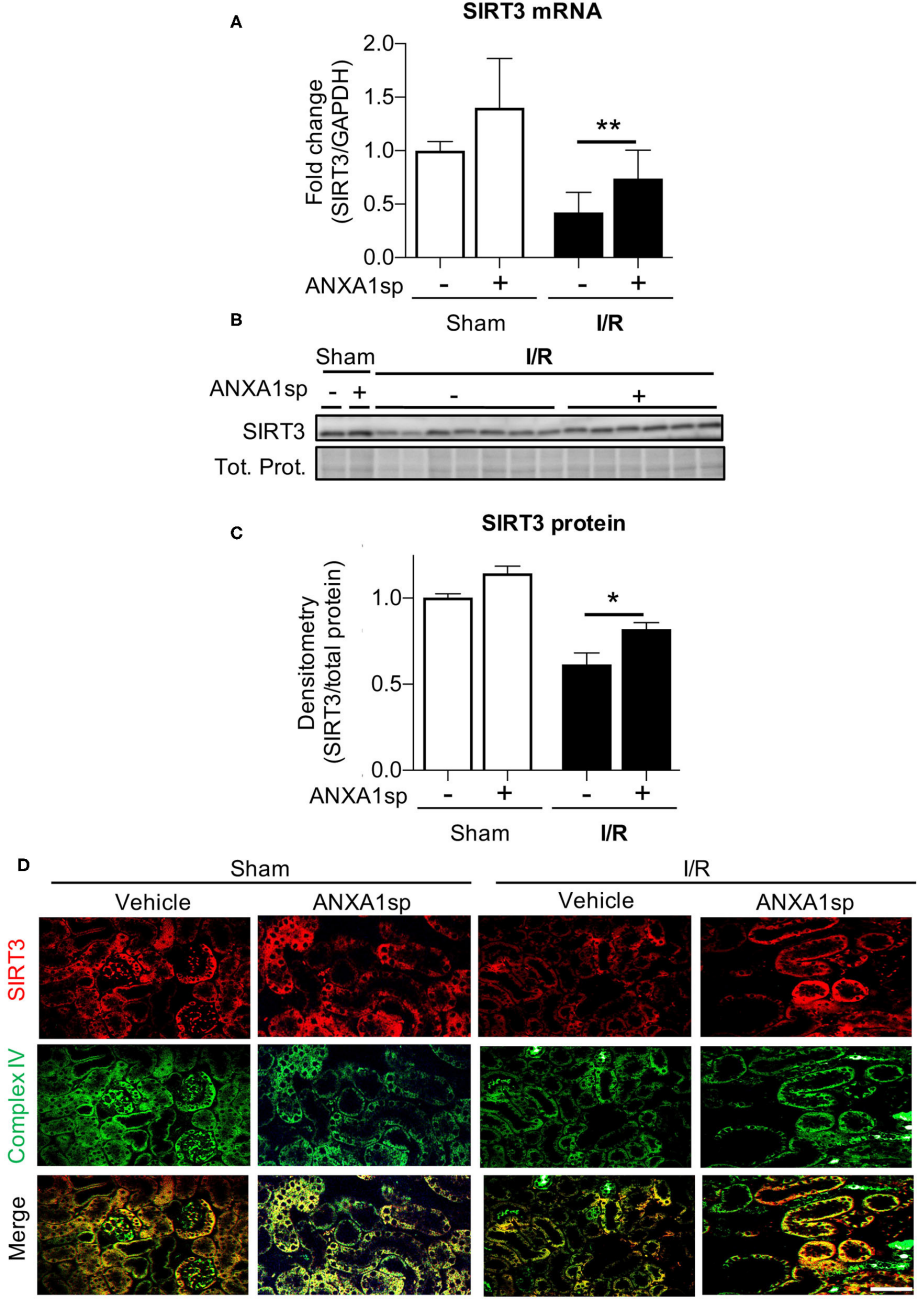
ANXA1sp treatment upregulates levels of sirtuin3 (SIRT3) in the mitochondria of kidney tubules. Mice were treated with either Vehicle or ANXA1sp 1 h prior to ischemia, subjected to 33 min of unilateral ischemia and contralateral nephrectomy, and then re-injected with Vehicle or ANXA1sp 1 h after reperfusion. Kidney tissues were harvested at 24 h after reperfusion. **(A)** mRNA levels of *SIRT3* mRNA were determined by RT-PCR. Graph displays mean +/-SEM of SIRT3 normalized to GADPH, then normalized to Sham, Vehicle group (*n* = 3 sample for Sham groups, *n* = 12 samples for I/R groups). Statistical significance determined by two-way ANOVA with Sidak post-test (^**^p < 0.01). **(B,C)** Protein levels of SIRT3 were determined by Western blot. Representative western blot in **(B)** with graph in **(C)** displaying mean +/-SEM of densitometry of SIRT3 normalized to total protein (Tot. Prot.) from stain-free gel for each sample, then normalized to Sham, Vehicle group (*n* = 3 samples for Sham groups, *n* = 6–7 for I/R groups). Statistical significance determined by two-way ANOVA with Sidak post-test (^*^p < 0.05). **(D)** Paraffin-embedded kidney tissue was analyzed for SIRT3 (top) and mitochondrial complex IV (middle) expression by immunofluorescence microscopy with merged image (bottom). SIRT3 co-localizes with mitochondrial marker complex IV in kidney tubule cells. Scale bar shows 50 μm.

Next, we wanted to determine the cellular mediators through which ANXA1sp and PGC1α could be working to protect mitochondria and prevent cell death. SIRT3 is a mitochondrial protectant that protects against kidney injury (Morigi et al., 2015; Zhao et al., 2018). SIRT3 is also a target gene of PGC1α (Kong et al., 2010). Thus, we next determined the effects of ANXA1sp on SIRT3 expression. Compared to vehicle-treated mice, we found that ANXA1sp upregulated SIRT3 at the mRNA and protein level after I/R (Figures 5A–C). By immunofluorescent staining, we also showed that SIRT3 expression appeared to be localized to mitochondria in kidney tubular cells as demonstrated by co-staining with mitochondrial complex IV (Figure 5D). We next measured acetylation of the SIRT3 target SOD2 to determine if ANXA1sp increased SIRT3 activity. We found increased acetylated SOD2 (reduced antioxidant activity) following I/R, which was reversed by ANXA1sp treatment (Supplementary Figure 3). We concluded that ANXA1sp promotes expression and activity of SIRT3 in mitochondria of kidney tubules following I/R, which likely helps to improve mitochondrial function following ischemia.

### ANXA1sp Prevents Kidney Tubular Cell Death Through SIRT3

**Figure 6:**
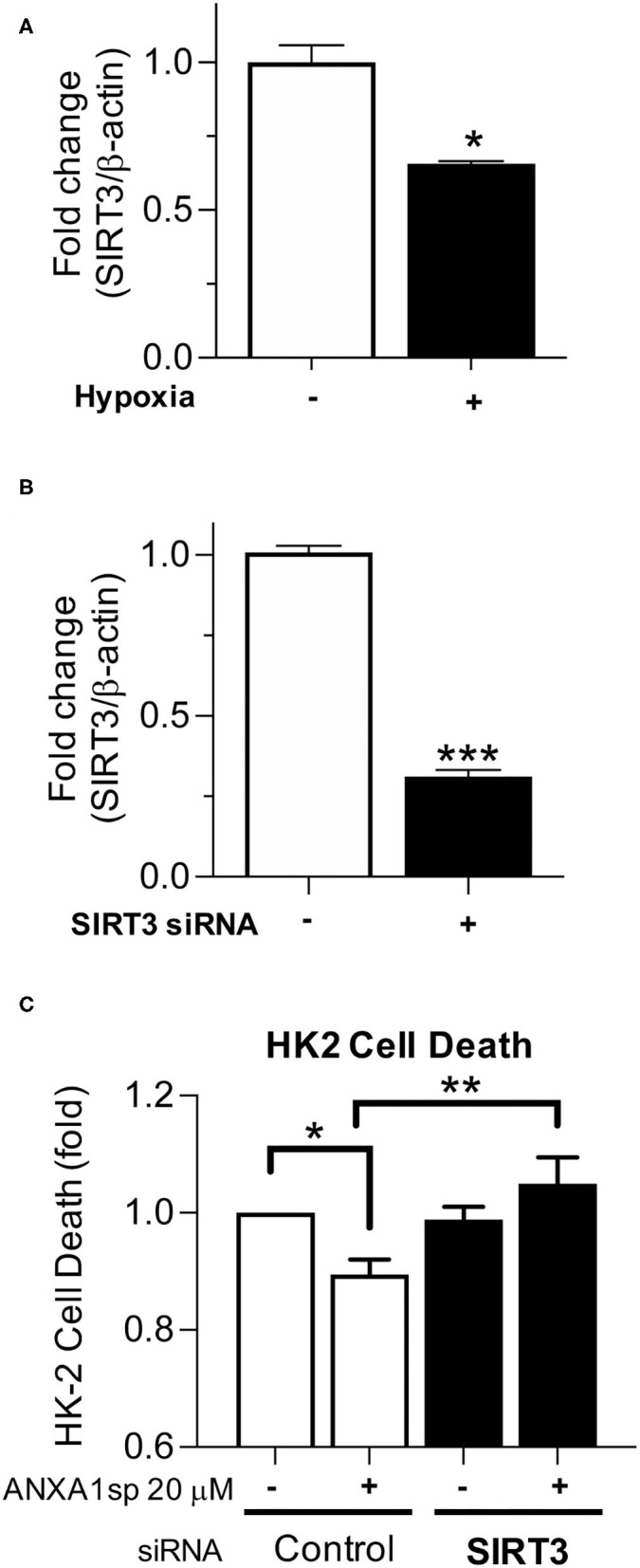
Silencing of SIRT3 prevents ANXA1sp-mediated protection from kidney cell death. **(A)** HK-2 cells were exposed to normoxic conditions or oxygen-glucose deprivation (Hypoxia) for 12 h and mRNA levels of SIRT3 were determined by RT-PCR. Graph displays mean +/- SEM (*n* = 3/group) with statistical significance determined by unpaired *T* test (^*^p < 0.05). **(B)** HK-2 cells were treated with control (-) or SIRT3 (+) siRNA. Levels of SIRT3 mRNA were determined by RT-PCR. Graph displays mean +/- SEM (*n* = 3/group) with statistical significance determined by unpaired *T*-test (^***^p < 0.001). **(C)** HK-2 were treated with control or SIRT3 siRNA. Cells were then pre-treated with Vehicle or ANXA1sp peptide for 1 h prior to oxygen-glucose deprivation for 16 h. Shown is fold change in cell death over Vehicle-treated, control siRNA cells. The protective effects of ANXA1sp were abrogated by SIRT3 siRNA (*n* = 6/condition). Graph displays mean +/- SEM with significance determined by one-way ANOVA with Sidak post-test (^*^p < 0.05, ^**^p < 0.01).

We next wanted to determine the necessity of SIRT3 to kidney cell death and ANXA1sp-mediated cellular protection *in vitro*. We found that similar to *in vivo* ischemia, hypoxia decreased SIRT3 gene transcription (Figure 6A). To determine whether SIRT3 was required for ANXA1sp-mediated amelioration of kidney tubular cell death, we next silenced SIRT3 expression with siRNA in HK-2 cells (Figure 6B) and exposed HK-2 cells to oxygen-glucose deprivation. We found that silencing of SIRT3 prevented ANXA1sp-mediated protection from hypoxic cell death (Figure 6C). Taken together, these findings demonstrate that ANXA1sp mediates its kidney protective effects through SIRT3 *in vitro*, which prevents kidney tubular cell death.

## Discussion

Our group has developed a tripeptide fragment, ANXA1sp, of the pro-resolving mediator annexin A1 that holds considerable promise for alleviating postoperative organ dysfunction. We hypothesized that ANXA1sp would alleviate AKI in a model of ischemic surgical kidney injury. In support of our hypothesis, we found that ANXA1sp limited post-surgical ischemic kidney injury. We showed that ANXA1sp treatment was associated with reduced kidney cell death following ischemia both *in vitro* and *in vivo*. We further demonstrated that ANXA1sp upregulated SIRT3 and limited markers of oxidative stress, induced markers of mitophagy and mitochondrial biogenesis, and limited mitochondrial fission, all of which attenuated cell death. In support of this mechanism of cellular protection, we further showed that the protective effects of ANXA1sp treatment were ameliorated when SIRT3 was silenced in kidney tubule cells *in vitro*. Taken together, ANXA1sp augments SIRT3 levels and improves markers associated with mitochondrial function, which ameliorates ischemic AKI.

AKI is a major cause of perioperative morbidity and mortality (Harris et al., 2015). Indeed, the kidney is one of the organs at a particularly increased risk of injury during the perioperative period. Since the kidneys receive nearly 25% of the cardiac output, they are continuously exposed to nephrotoxins during surgery (Zarbock et al., 2018; Gumbert et al., 2020); and hemodynamic alterations, including hypotension and hypovolemia, place the kidneys at an additional risk for injury. Surgeries performed on the aorta, such as abdominal aortic repair, require clamping of the abdominal aorta and/or renal vessels, which directly induces ischemic injury. Likewise, in kidney transplantation, the renal vessels are clamped to allow the removal of the donor kidney. During all of these procedures, the timing of kidney insult is known. As such, the identification of kidney-protective therapeutics that can be given prior to a known kidney insult holds considerable promise for limiting perioperative kidney injury. Based on our results here, we believe ANXA1sp shows promise as a perioperative kidney protectant. In addition, though it remains to be tested, since transplanted donor kidneys undergo a period of ischemia between harvest and transplant, ANXA1sp could also be used to preserve donor kidneys prior to transplantation.

The efficacy of ANXA1sp to limit organ injury in the brain (Zhang et al., 2017; Ma et al., 2019), and now the kidney, points to its mechanism of protection being modulation of a global cellular function. One possibility we considered could be limiting inflammation as the parent annexin A1 molecule is known to reduce leukocyte tethering and transmigration and promote phagocytosis of dead cells (Mancuso et al., 1995; Arur et al., 2003; De Coupade et al., 2003; Scannell et al., 2007). However, as ANXA1sp has shown promise to limit cell death in the brain (Zhang et al., 2017; Ma et al., 2019), we tested the ability of ANXA1sp to limit cell death in the kidney following ischemia. We indeed found that ANXA1sp was able to mitigate cell death following ischemia in the kidney and in immortalized human kidney cell lines in an *in vitro* model of hypoxia. Taken together with its effects on limiting cell death in the brain, ANXA1sp appears to broadly limit cell death in multiple tissues.

ANXA1sp appears to limit cell death through SIRT3-mediated mitochondrial protection. The sirtuins are a family of seven NAD^+^-dependent deacetylases (SIRT1-7) that are known to mitigate cellular stress and improve cellular metabolic function (Hershberger et al., 2017). We focused our attention on SIRT3 as we found that ANXA1sp administration was associated with markers of improved mitochondrial function, mitochondrial biogenesis, mitophagy, and decreased oxidative stress—all processes that SIRT3 is known to regulate (Meng et al., 2019). Moreover, at least *in vitro*, the protective effects of ANXA1sp were abolished when SIRT3 was silenced, implying ANXA1sp mediates its kidney protective effects through SIRT3. We do recognize that limitations of our study include the lack of direct evidence that SIRT3 is required for the protective effects of ANXA1sp in the kidneys of mice *in vivo*, and that our *in vitro* studies used immortalized human proximal tubular cell lines. However, our data showing augmented SIRT3 expression and de-acetylation of SIRT3 targets is in line with other groups that have shown that SIRT3 deletion is deleterious in both nephrotoxic and septic AKI (Morigi et al., 2015; Zhao et al., 2018). ANXA1sp could modulate its effects through other sirtuin family members; however, it is less likely that ANXA1sp is mediating its effects through SIRT1, SIRT2, SIRT6, or SIRT7 as their subcellular localization is outside the mitochondria (Hershberger et al., 2017). The possibility does remain, however, that ANXA1sp could upregulate SIRT3 levels indirectly through SIRT1 as SIRT1 is a known activator of PGC-1α (Rodgers et al., 2005), SIRT3 is thought to be regulated at the transcriptional level by PGC1α (Kong et al., 2010), and we show upregulation of PGC1α following ANXA1sp treatment. ANXA1sp could also regulate the other mitochondrial sirtuins, SIRT4 and SIRT5, although the effects of these sirtuin family members in AKI is less well-established. The effects of ANXA1sp on other sirtuin family members and their role in AKI remain to be determined.

Owing to its mitochondrial protective properties, ANXA1sp appears to be particularly efficacious at limiting cell stress in tissues with a high mitochondrial content. Due to the high metabolic demands required to remove waste products and regulate acid-base status, fluids, electrolytes, and blood pressure, the kidney is one of the most metabolically active organs in the body. In fact, of all the organs, the kidney has the second highest mitochondrial content and oxygen consumption after the heart (O'connor, 2006; Pagliarini et al., 2008). Damaged mitochondria increase ROS production, propagating mitochondrial damage (Giorgi et al., 2018), which may stimulate the release of pro-cell death factors, such as cytochrome c, from mitochondria into cytosol to initiate cell death (Tang et al., 2015). Ischemia-reperfusion events are frequently complicated by mitochondrial dysfunction and increased ROS production. We showed that ANXA1sp limits cell death and increases antioxidant enzymes. ANXA1sp suppression of oxidative stress in the kidney is again likely due to its upregulation of SIRT3 (Pillai et al., 2010): We show that ANXA1sp upregulates OGG1, an important mitochondrial DNA repair enzyme, and SIRT3 can interact with OGG1 (Cheng et al., 2013). In addition, under stress conditions, ROS production can exceed the antioxidant capacity of the mitochondria due to acetylation-induced inactivation of mitochondrial antioxidant enzymes SOD2, which is deacetylated and activated by SIRT3 (Fernandez-Marcos et al., 2012). We show both an upregulation and de-acetylation of the SIRT3 target SOD2. Thus, in addition to the direct upregulation of SOD2 expression that we show here, ANXA1sp-mediated upregulation of SIRT3 also likely augments the activation of SOD2 to limit ROS damage. Taken together, ANXA1sp augments the mitochondrial antioxidant system, which likely helps to prevent ROS-induced mtDNA oxidation and damage. Furthermore, Tfam is a major replication and transcription factor of mtDNA, and SIRT3 deacetylates Tfam and enhances its activity (Hebert et al., 2013), which may help explain the increase of mitochondrial biogenesis seen in ANXA1sp treated mice.

ANXA1sp also improves markers of mitophagy, a selective form of autophagy that eliminates redundant or damaged mitochondria (Harper et al., 2018). Recent evidence suggests that mitophagy plays an important role in AKI development and subsequent kidney repair: PINK1-Parkin-mediated mitophagy was reported to be protective against both toxic and ischemic kidney injury (Tang et al., 2018; Wang et al., 2018). Not only did ANXA1sp treatment improve markers of mitophagy in our studies, but it also reduced levels of the mitochondrial fission marker Drp1, suggesting that ANXA1sp limits mitochondrial fragmentation. The influence of Drp1 and mitochondrial fragmentation on apoptosis and exacerbation of injury has been documented in several studies (Brooks and Dong, 2007; Brooks et al., 2009, 2011), including in the kidney (Perry et al., 2018). Thus, inhibiting this response likely protects the kidney from further injury after ischemia. The specific mechanism by which ANXA1sp decreases mitochondrial-associated Drp1 is not known; however, activation of the SIRT3 and mitophagy program may also influence mitochondrial dynamics after ischemia.

The mechanisms by which ANXA1sp upregulates SIRT3 to protect the mitochondria are still unclear. The parent annexin A1 molecule binds the formyl peptide receptor 2 (FPR2), a promiscuous G-protein coupled receptor (GPCR) that serves as a pattern recognition receptor for bacterial formylpeptides, eicosanoid lipid molecules, and pro-resolving lipid mediators (Zhuang et al., 2020). As ANXA1sp is a small tripeptide fragment of the parent annexin A1 molecule, it is likely that ANXA1sp is able to bind to and activate FPR2. However, due to its small size, ANXA1sp may have multiple cellular targets. The identification of the cellular target of ANXA1sp is an active area of investigation in our laboratory.

In conclusion, the annexin A1 mimetic peptide, ANXA1sp, upregulates SIRT3 and augments markers associated with improved mitochondrial function in the kidney following ischemic injury. Restoration of mitochondrial function via ANXA1sp/SIRT3 may offer unique pharmacological targets for improved recovery from AKI. Thus, ANXA1sp holds considerable promise as a perioperative kidney protectant.

## Data Availability Statement

The raw data supporting the conclusions of this article will be made available by the authors, without undue reservation.

## Ethics Statement

The animal study was reviewed and approved by Durham Veterans Affairs Medical Center (VAMC) Institutional Animal Care and Use Committee.

## Author Contributions

HS helped with the study design, conducting the experiments, acquiring the data, providing the reagents, and writing/editing the manuscript. QM helped with the study design, conducting the experiments, acquiring the data, and editing the manuscript. ZZ helped with the study design, conducting the experiments, acquiring the data, providing the reagents, and editing the manuscript. JR helped with acquiring the data and editing the manuscript. BM helped with conducting the experiments, acquiring the data, and editing the manuscript. SC helped with the study design, providing the reagents, and editing the manuscript. LU helped with the study design and editing manuscript. JP helped with the study design, conducting the experiments, acquiring the data, providing the reagents, and writing the manuscript. All authors contributed to the article and approved the submitted version.

## Conflict of Interest

ZZ and QM are coinventors on patents filed through Duke University on the therapeutic use of Annexin A1 tripeptide (ANXA1sp). The remaining authors declare that the research was conducted in the absence of any commercial or financial relationships that could be construed as a potential conflict of interest.
