# Supplementary material for: Annexin A1 Tripeptide Mimetic Increases Sirtuin-3 and Augments Mitochondrial Function to Limit Ischemic Kidney Injury
Source: Front Physiol. 2021 Jul 1;12:683098. doi: 10.3389/fphys.2021.683098 (PMC8281307; doi:10.3389/fphys.2021.683098)
Supplement: Supplementary file 1 [file Data_Sheet_1.docx]

Supplementary Material


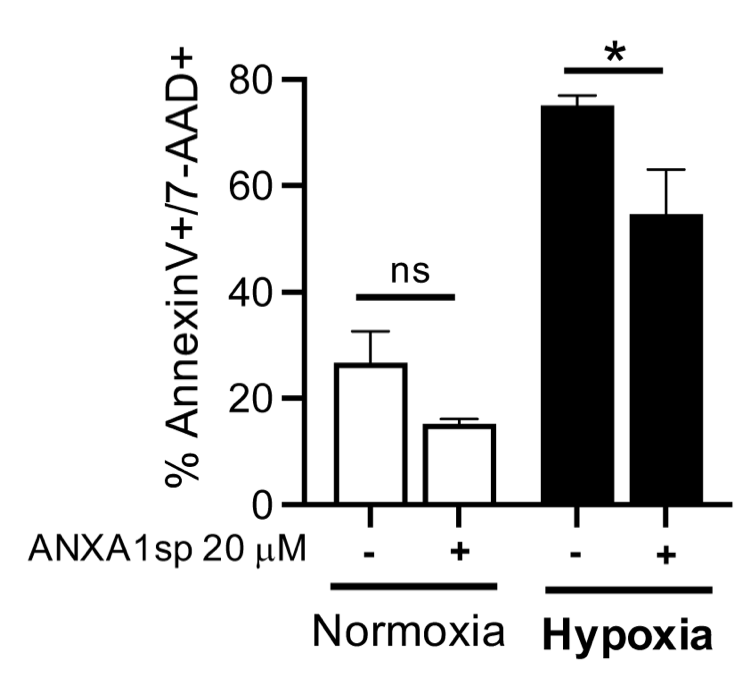


**Supplemental Figure 1 – ANXA1sp treatment limits cell death in immortalized human renal proximal tubular cells following hypoxia.** The immortalized human renal proximal tubular epithelial cell line, RPTEC/TERT1, was grown to confluence in monolayers. Cells were pretreated with Vehicle or ANXA1sp (20μM) and then subjected to 14 hours of oxygen-glucose deprivation (hypoxia) in an anaerobic chamber. ANXA1sp prevented hypoxic cell death (n=3/condition). Graphs display mean +/- SEM with significance determined by two-way ANOVA with Sidak post-test (*p<0.05).

**Supplemental Figure 2 – ANXA1sp treatment promotes mitochondrial biogenesis.** Mice were treated with either Vehicle or ANXA1sp 1 hour prior to ischemia, subjected to 33 minutes of unilateral ischemia and contralateral nephrectomy and then re-injected with Vehicle or ANXA1sp 1 hour after reperfusion. Kidney tissues were harvested at 24 hours after reperfusion. (A)Western blot showing protein levels of PGC1α with densitometry shown in (B). (C) Western blot showing indicated proteins with densitometry results for (D) mitochondrial citrate synthase (mtCS), (E) mitochondrial complex I (mtCI), and (F) mitochondrial NADH-ubiquinone oxidoreductase chain 1 (mtND1). Graphs display mean +/-SEM of densitometry of mitochondrial protein normalized to porin. Statistical significance determined by two-way ANOVA (n=3 samples for Sham groups, n=6 samples for I/R groups; *p<0.05, **p<0.01).

**Supplemental Figure 3 - ANXA1sp decreases acetylation of SOD2**. Mice were treated with either Vehicle or ANXA1sp 1 hour prior to ischemia, subjected to 33 minutes of unilateral ischemia and contralateral nephrectomy (I/R) and then re-injected with Vehicle or ANXA1sp 1 hour after reperfusion. Kidney tissues were harvested at 24 hours after reperfusion. (A) SOD2 was immunoprecipitated (IP) from kidney tissue lysates and immunoblot (IB) performed for acetylated lysine (Ac-Lys) residues. (B) ANXA1sp decreases SOD2 acetylation and activates SOD2. Graph displays mean +/- SEM (n = 3/groups; statistics determined by two-way ANOVA, ** p<0.01, ***p<0.001).
